# Disrupting Electronic Health Records Systems: The Next Generation

**DOI:** 10.2196/medinform.4192

**Published:** 2015-10-23

**Authors:** Leo Anthony Celi, Jeffrey David Marshall, Yuan Lai, David J Stone

**Affiliations:** ^1^ Beth Israel Deaconess Medical Center Division of Pulmonary, Critical Care, and Sleep Medicine Boston, MA United States; ^2^ Yale University, Yale-New Haven Hospital Department of Pulmonary and Critical Care New Haven, CT United States; ^3^ Center for Urban Science and Progress New York University New York, NY United States; ^4^ Center for Wireless Health Departments of Anesthesiology and Neurological Surgery University of Virginia Charlottesville, VA United States

**Keywords:** clinical decision making, clinical decision support, electronic health records, electronic notes

## Abstract

The health care system suffers from both inefficient and ineffective use of data. Data are suboptimally displayed to users, undernetworked, underutilized, and wasted. Errors, inefficiencies, and increased costs occur on the basis of unavailable data in a system that does not coordinate the exchange of information, or adequately support its use. Clinicians’ schedules are stretched to the limit and yet the system in which they work exerts little effort to streamline and support carefully engineered care processes. Information for decision-making is difficult to access in the context of hurried real-time workflows. This paper explores and addresses these issues to formulate an improved design for clinical workflow, information exchange, and decision making based on the use of electronic health records.

## Introduction

Weed introduced the “Subjective, Objective, Assessment, and Plan” (SOAP) note in the late 1960s [1]. This note entails a high-level structure that supports the thought process that goes into decision-making: subjective data followed by ostensibly more reliable objective data employed to formulate an assessment and subsequent plan. The flow of information has not fundamentally changed since that time, but the complexities of the information, possible assessments, and therapeutic options certainly have greatly expanded. Clinicians have not heretofore created anything like an optimal data system for medicine [2,3]. Such a system is essential to streamline workflow and support decision-making rather than adding to the time and frustration of documentation [4].

What this optimal data system offers is not a radical departure from the traditional thought processes that go into the production of a thoughtful and useful note. However, in the current early stage digitized medical system, it is still incumbent on the decision maker/note creator to capture the relevant priors, and to some extent, digitally scramble to collect all the necessary updates. The capture of these priors is a particular challenge in an era where care is more frequently turned over among different caregivers than ever before. Finally, based on a familiarity of the disease pathophysiology, the medical literature and evidence-based medicine (EBM) resources, the user is tasked with creating an optimal plan based on that assessment. In this so-called digital age, the amount of memorization, search, and assembly can be minimized and positively supported by a well-engineered system purposefully designed to assist clinicians in note creation and, in the process, decision-making.

**Figure 1:**
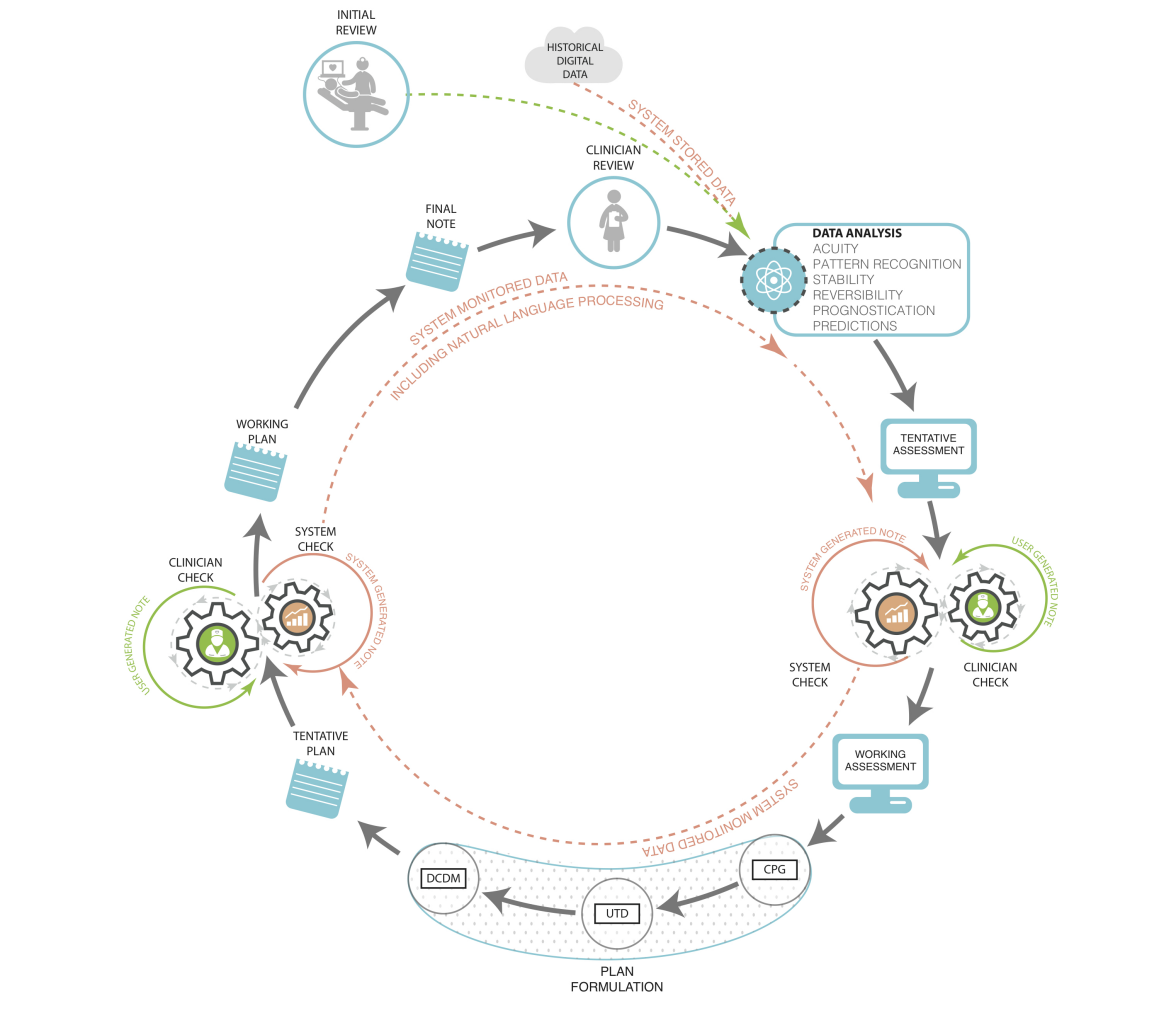
Clinician documentation with fully integrated data systems support. Prior notes and data are input for the following note and decisions. Machine analyzes input and displays suggested diagnoses and problem list, and test and treatment recommendations based on various levels of evidence: CPG – clinical practice guidelines, UTD – Up to Date®, DCDM – Dynamic Clinical Data Mining.

Since 2006, use of electronic health records (EHRs) by US physicians increased by over 160% with 78% of office-based physicians and 59% of hospitals having adopted an EHR by 2013 [5,6]. With implementation of federal incentive programs, a majority of EHRs were required to have some form of built-in clinical decision support tools by the end of 2012 with further requirements mandated as the *Affordable Care Act* (ACA) rolls out [7]. These requirements recognize the growing importance of standardization and systematization of clinical decision-making in the context of the rapidly changing, growing, and advancing field of medical knowledge. There are already EHRs and other technologies that exist, and some that are being implemented, that integrate clinical decision support into their functionality, but a more intelligent and supportive system can be designed that capitalizes on the note writing process itself. We should strive to optimize the note creation process as well as the contents of the note in order to best facilitate communication and care coordination. The following sections characterize the elements and functions of this decision support system (Figure 1).

## Incorporating Data

Overwhelmingly, the most important characteristic of the electronic note is its potential for the creation and reception of what we term “bidirectional data streams” to inform both decision-making and research. By bidirectional data exchange, we mean that electronic notes have the potential to provide data streams to the entirety of the EHR database and vice versa*.* The data from the note can be recorded, stored, accessed, retrieved, and mined for a variety of real-time and future uses. This process should be an automatic and intrinsic property of clinical information systems. The incoming data stream is currently produced by the data that is slated for import into the note according to the software requirements of the application and the locally available interfaces [8]. The provision of information from the note to the system has both short- and long-term benefits: in the short term, this information provides essential elements for functions such as benchmarking and quality reporting; and in the long term, the information provides the afferent arm of the learning system that will identify individualized best practices that can be applied to individual patients in future formulations of plans.

Current patient data should include all the electronically interfaced elements that are available and pertinent. In addition to the usual elements that may be imported into notes (eg, laboratory results and current medications), the data should include the immediate prior diagnoses and treatment items, so far as available (especially an issue for the first note in a care sequence such as in the ICU), the active problem list, as well as other updates such as imaging, other kinds of testing, and consultant input. Patient input data should be included after verification (eg, updated reviews of systems, allergies, actual medications being taken, past medical history, family history, substance use, social/travel history, and medical diary that may include data from medical devices). These data priors provide a starting point that is particularly critical for those note writers who are not especially (or at all) familiar with the patient. They represent historical (and yet dynamic) *evidence* intended to inform decision-making rather than "text" to be thoughtlessly carried forward or copied and pasted into the current note.

Although the amount and types of data collected are extremely important, how it is used and displayed are paramount. Many historical elements of note writing are inexcusably costly in terms of clinician time and effort when viewed at a level throughout the entire health care system. Redundant items such as laboratory results and copy-and-pasted nursing flow sheet data introduce a variety of “chartjunk” that clutters documentation and makes the identification of truly important information more difficult and potentially even introduces errors that are then propagated throughout the chart [9,10]. Electronic systems are poised to automatically capture the salient components of care so far as these values are interfaced into the system and can even generate an active problem list for the providers. With significant amounts of free text and “unstructured data” being entered, EHRs will need to incorporate more sophisticated processes such as natural language processing and machine learning to provide accurate interpretation of text entered by a variety of different users, from different sources, and in different formats, and then translated into structured data that can be analyzed by the system.

Optimally, a fully functional EHR would be able to provide useful predictive data analytics including the identification of patterns that characterize a patient’s normal physiologic state (thereby enabling detection of significant change from that state), as well as mapping of the predicted clinical trajectory, such as prognosis of patients with sepsis under a number of different clinical scenarios, and with the ability to suggest potential interventions to improve morbidity or mortality [11]. Genomic and other “-omic” information will eventually be useful in categorizing certain findings on the basis of individual susceptibilities to various clinical problems such as sepsis, auto-immune disease, and cancer, and in individualizing diagnostic and treatment recommendations. In addition, an embedded data analytic function will be able to recognize a constellation of relatively subtle changes that are difficult or impossible to detect, especially in the presence of chronic co-morbidities (eg, changes consistent with pulmonary embolism, which can be a subtle and difficult diagnosis in the presence of long standing heart and/or lung disease) [12,13].

The data presentation section must be thoughtfully displayed so that the user is not overwhelmed, but is still aware of what elements are available, and directed to those aspects that are most important. The user then has the tools at hand to construct the truly cognitive sections of the note: the assessment and plan. Data should be displayed in a fashion that efficiently and effectively provides a maximally informationally rich and minimally distracting graphic display. The fundamental principle should result in a thoughtfully planned data display created on the ethos of “just enough and no more,” as well as the incorporation of clinical elements such as severity, acuity, stability, and reversibility. In addition to the now classic teachings of Edward Tufte in this regard, a number of new data artists have entered the field [14]. There is room for much innovation and improvement in this area, as medicine transitions from paper to a digital format that provides enormous potential and capability for new types of displays.

## Integrating the Monitors

Bedside and telemetry monitoring systems have become an element of the clinical information system but they do not yet interact with the EHR in a bidirectional fashion to provide decision support. In addition to the raw data elements, the monitors can provide data analytics that could support real-time clinical assessment as well as material for predictive purposes apart from the traditional noisy alarms [15,16]. It may be less apparent how the reverse stream (EHR to bedside monitor) would work, but the EHR can set the context for the interpretation of raw physiologic signals based on previously digitally captured vital signs, patient co-morbidities and current medications, as well as the acute clinical context.

In addition, the display could provide an indication of whether technically ”out of normal range” vital signs (or labs in the emergency screen described below) are actually “abnormal” for this particular patient. For example, a particular type of laboratory value for a patient may have been chronically out of normal range and not represent a change requiring acute investigation and/or treatment. This might be accomplished by displaying these types of ”normally abnormal” values in purple or green rather than red font for abnormal, or via some other designating graphic. The purple font (or whatever display mode was utilized) would designate the value as technically abnormal, but perhaps not *contextually* abnormal. Such designations are particularly important for caregivers who are not familiar with the patient.

It also might be desirable to use a combination of accumulated historical data from the monitor and the EHR to formulate personalized alarm limits for each patient. Such personalized alarm limits would provide a smarter range of acceptable values for each patient and perhaps also act to reduce the unacceptable number of false positive alarms that currently plague bedside caregivers (and patients) [17]. These alarm limits would be dynamically based on the input data and subject to reformulation as circumstances changed. We realize that any venture into alarm settings becomes a regulatory and potentially medico-legal issue, but these intimidating factors should not be allowed to grind potentially beneficial innovations to a halt. For example, “hard” limits could be built into the alarm machine so that the custom alarm limits could not fall outside certain designated values.

## Supporting the Formulation of the Assessment

Building on both prior and new, interfaced and manually entered data as described above, the next framework element would consist of the formulation of the note in real time. This would consist of structured data so far as available and feasible, but is more likely to require real-time natural language processing performed on the free text being entered. Different takes on this kind of templated structure have already been introduced into several electronic systems. These include note templates created for specific purposes such as end-of-life discussions, or documentation of cardiopulmonary arrest. The very nature of these note types provides a robust context for the content. We also recognize that these shorter and more directed types of notes are not likely to require the kind of extensive clinical decision support (CDS) from which an admission or daily progress note may benefit.

Until the developers of EHRs find a way to fit structured data selection seamlessly and transparently into workflow, we will have to do the best we can with the free text that we have available. While this is a bit clunky in terms of data utilization purposes, perhaps it is not totally undesirable, as free text inserts a needed narrative element into the otherwise storyless EHR environment. Medical care can be described as an ongoing story and free text conveys this story in a much more effective and interesting fashion than do selected structured data bits. Furthermore, stories tend to be more distinctive than lists of structured data entries, which sometimes seem to vary remarkably little from patient to patient. But to extract the necessary information, the computer still needs a processed interpretation of that text. More complex systems are being developed and actively researched to act more analogously to our own ”human” form of clinical problem solving [18], but until these systems are integrated into existing EHRs, clinicians may be able to help by being trained to minimize the potential confusion engendered by reducing completely unconstrained free text entries and/or utilizing some degree of standardization within the use of free text terminologies and contextual modifiers.

**Figure 2:**
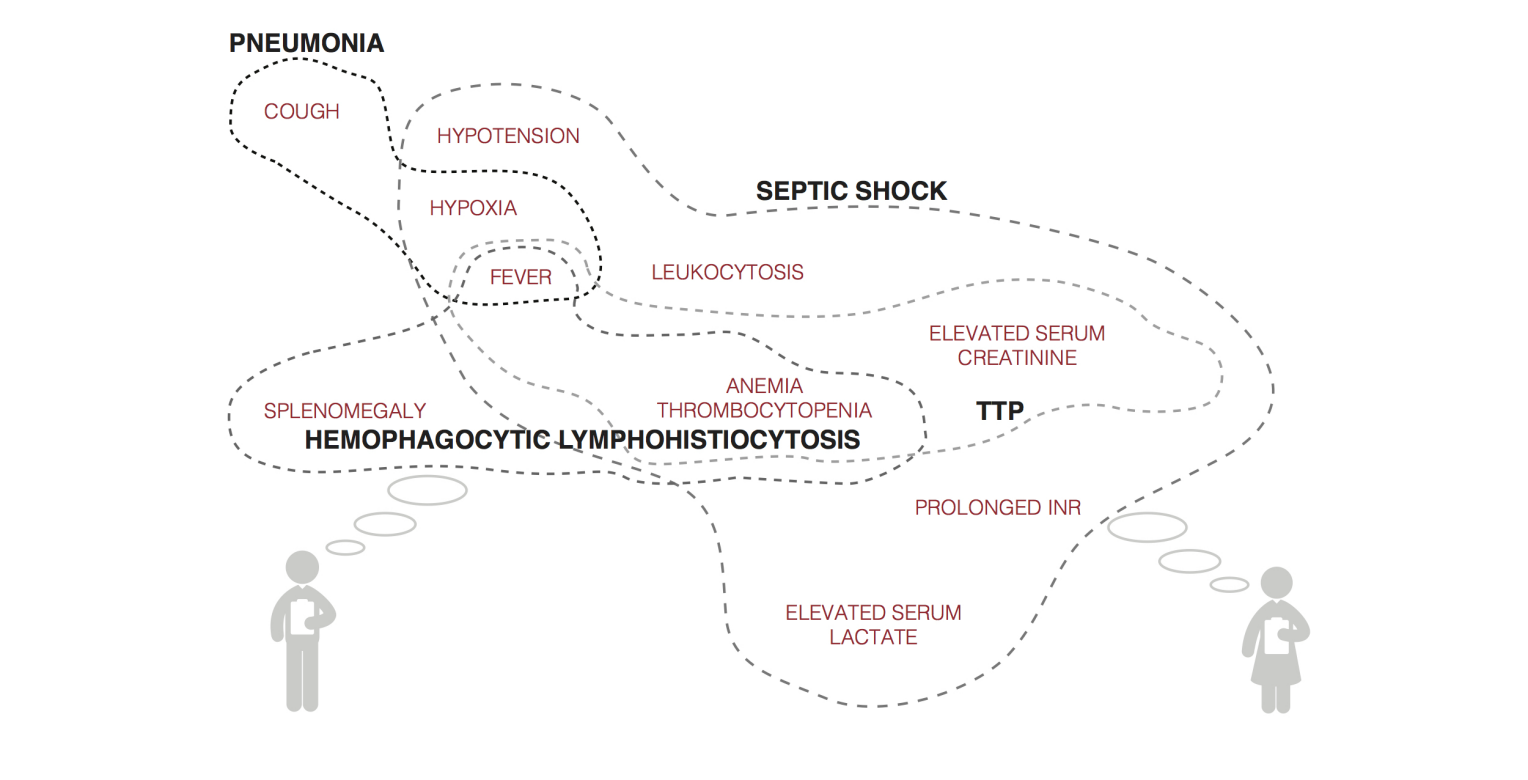
Mock visualization of symptoms, signs, laboratory results, and other data input and systems suggestion for differential diagnoses.

Employing the prior data (eg, diagnoses X, Y, Z from the previous note) and new data inputs (eg, laboratory results, imaging reports, and consultants’ recommendations) in conjunction with the assessment being entered, the system would have the capability to check for inconsistencies and omissions based on analysis of both prior and new entries. For example, a patient in the ICU has increasing temperature and heart rate, and decreasing oxygen saturation. These continuous variables are referenced against other patient features and risk factors to suggest the possibility that the patient has developed a pulmonary embolism or an infectious ventilator-associated complication. The system then displays these possible diagnoses within the working assessment screen with hyperlinks to the patient’s flow sheets and other data supporting the suggested problems (Figure 2). The formulation of the assessment is clearly not as potentially evidence-based as that of the plan; however, there should still be dynamic, automatic and rapid searches performed for pertinent supporting material in the formulation of the assessment. These would include the medical literature, including textbooks, online databases, and applications such as WebMD. The relevant literature that the system has identified, supporting the associations listed in the assessment and plan*,* can then be screened by the user for accuracy and pertinence to the specific clinical context. Another potentially useful CDS tool for assessment formulation is a modality we have termed dynamic clinical data mining (DCDM) [19]. DCDM draws upon the power of large sets of population health data to provide differential diagnoses associated with groupings or constellations of symptoms and findings. Similar to the process just described, the clinician would then have the ability to review and incorporate these suggestions or not.

An optional active search function would also be provided throughout the note creation process for additional flexibility—clinicians are already using search engines, but doing so sometimes in the absence of specific clinical search algorithms (eg, a generic search engine such Google). This may produce search results that are not always of the highest possible quality [20,21]. The EHR-embedded search engine would have its algorithm modified to meet the task as Google has done previously for its search engine [22]. The searchable TRIP database provides a search engine for high-quality clinical evidence, as do the search modalities within Up to Date, Dynamed, BMJ Clinical Evidence, and others [23,24].

## Supporting the Formulation of the Plan

With the assessment formulated, the system would then formulate a proposed plan using EBM inputs and DCDM refinements for issues lying outside EBM knowledge. Decision support for plan formulation would include items such as randomized control trials (RCTs), observational studies, clinical practice guidelines (CPGs), local guidelines, and other relevant elements (eg, Cochrane reviews). The system would provide these supporting modalities in a hierarchical fashion using evidence of the highest quality first before proceeding down the chain to lower quality evidence. Notably, RCT data are not available for the majority of specific clinical questions, or it is not applicable because the results cannot be generalized to the patient at hand due to the study’s inclusion and exclusion criteria [25]. Sufficiently reliable observational research data also may not be available, although we expect that the holes in the RCT literature will be increasingly filled by observational studies in the near future [16,26]. In the absence of pertinent evidence-based material, the system would include the functionality which we have termed DCDM, and our Stanford colleagues have termed the “green button” [19,27]. This still-theoretical process is described in detail in the references, but in brief, DCDM would utilize a search engine type of approach to examine a population database to identify similar patients on the basis of the information entered in the EHR. The prior treatments and outcomes of these historical patients would then be analyzed to present options for the care of the current patient that were, to a large degree, based on prior data. The efficacy of DCDM would depend on, among other factors, the availability of a sufficiently large population EHR database, or an open repository that would allow for the sharing of patient data between EHRs. This possibility is quickly becoming a reality with the advent of large, deidentified clinical databases such as that being created by the Patient Centered Outcomes Research Institute [26].

**Figure 3:**
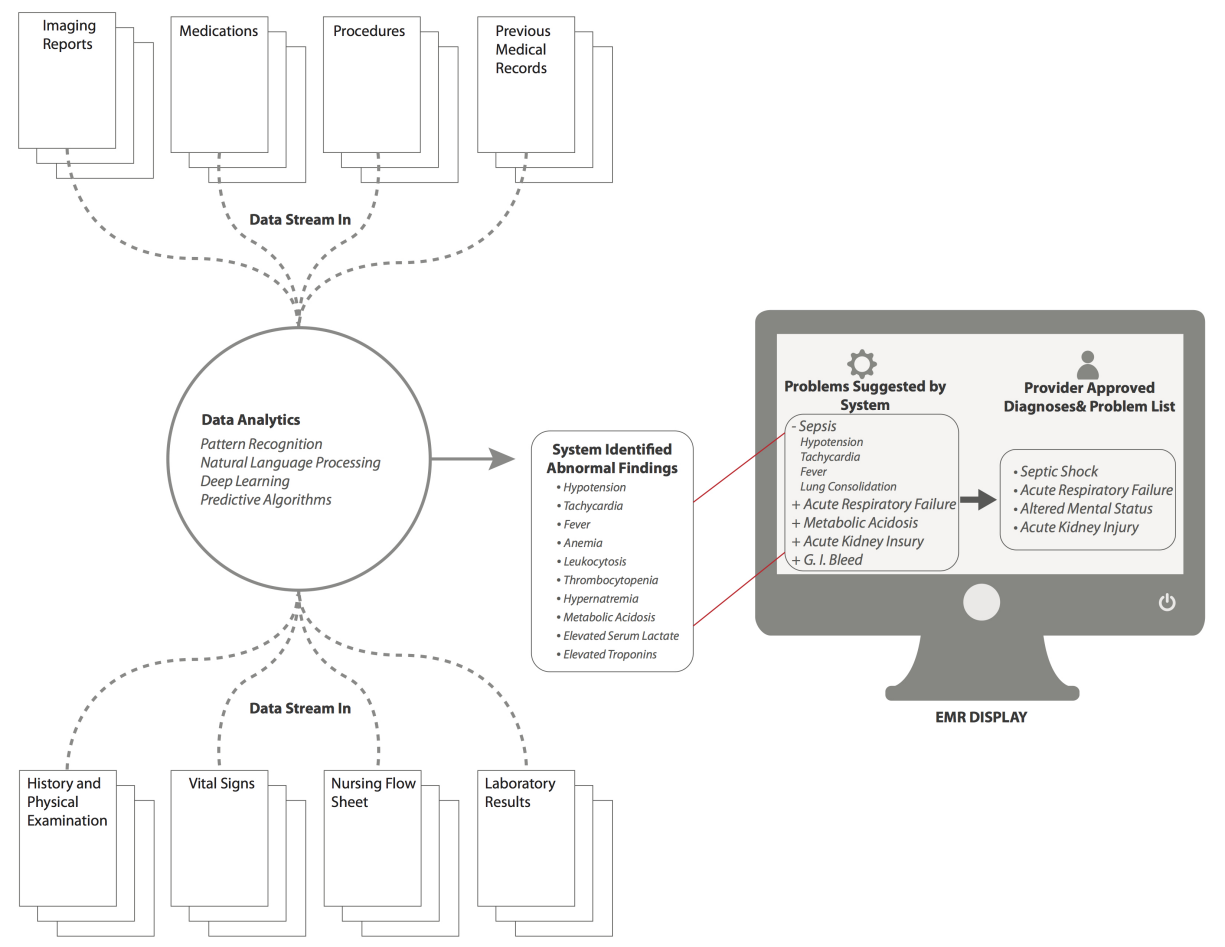
Mock screenshot for the "Assessment and Plan" screen with background data analytics. Based on background analytics that are being run by the system at all times, a series of "problems" are identified and suggested by the system, which are then displayed in the EMR in the box on the left. The clinician can then select problems that are suggested, or input new problems that are then displayed in the the box on the right of the EMR screen, and will now be apart of ongoing analytics for future assessment.

The tentative plan could then be modified by the user on the basis of her or his clinical "wetware" analysis. The electronic workflow could be designed in a number of ways that were modifiable per user choice/customization. For example, the user could first create the assessment and plan which would then be subject to comment and modification by the automatic system. This modification might include suggestions such as adding entirely new items, as well as the editing of entered items. In contrast, as described, the system could formulate an original assessment and plan that was subject to final editing by the user. In either case, the user would determine the final output, but the system would record both system and final user outputs for possible reporting purposes (eg, consistency with best practices). Another design approach might be to display the user entry *in toto* on the left half of a computer screen and a system-formulated assessment (Figure 3) and plan on the right side for comparison. Links would be provided throughout the system formulation so that the user could drill into EHR-provided suggestions for validation and further investigation and learning. In either type of workflow, the system would comparatively evaluate the final entered plan for consistency, completeness, and conformity with current best practices. The system could display the specific items that came under question and why. Users may proceed to adopt or not, with the option to justify their decision. Data reporting analytics could be formulated on the basis of compliance with EBM care. Such analytics should be done and interpreted with the knowledge that EBM itself is a moving target and many clinical situations do not lend themselves to resolution with the current tools supplied by EBM.

Since not all notes call for this kind of extensive decision support, the CDS material could be displayed in a separate columnar window adjacent to the main part of the screen where the note contents were displayed so that workflow is not affected. Another possibility would be an “opt-out” button by which the user would choose not to utilize these system resources. This would be analogous but functionally opposite to the "green button" opt*-in* option suggested by Longhurst et al, and perhaps be designated the "orange button" to clearly make this distinction [27]. Later, the system would make a determination as to whether this lack of EBM utilization was justified, and provide a reminder if the care was determined to be outside the bounds of current best practices. While the goal is to keep the user on the EBM track as much as feasible, the system has to "realize" that real care will still extend outside those bounds for some time, and that some notes and decisions simply do not require such machine support.

There are clearly still many details to be worked out regarding the creation and use of a fully integrated *bidirectional* EHR. There currently are smaller systems that use some components of what we propose. For example, a large Boston hospital uses a program called QPID which culls all previously collected patient data and uses a Google-like search to identify specific details of relevant prior medical history which is then displayed in a user-friendly fashion to assist the clinician in making real-time decisions on admission [28]. Another organization, the American Society of Clinical Oncology, has developed a clinical Health IT tool called CancerLinQ which utilizes large clinical databases of cancer patients to trend current practices and compare the specific practices of individual providers with best practice guidelines [29]. Another hospital system is using many of the components discussed in a new, internally developed platform called Fluence that allows aggregation of patient information, and applies already known clinical practice guidelines to patients’ problem lists to assist practitioners in making evidenced-based decisions [30]. All of these efforts reflect inadequacies in current EHRs and are important pieces in the process of selectively and wisely incorporating these technologies into EHRs, but doing so universally will be a much larger endeavor.

## Conclusions

Medicine has finally entered an era in which clinical digitization implementations and data analytic systems are converging. We have begun to recognize the power of data in other domains and are beginning to apply it to the clinical space, applying digitization as a necessary but insufficient tool for this purpose (personal communication from Peter Szolovits, *The Unreasonable Effectiveness of Clinical Data. Challenges in Big Data for Data Mining, Machine Learning and Statistics Conference, March 2014*). The vast amount of information and clinical choices demands that we provide better supports for making decisions and effectively documenting them. The Institute of Medicine demands a “learning health care system” where analysis of patient data is a key element in continuously improving clinical outcomes [31]. This is also an age of increasing medical complexity bound up in increasing financial and time constraints. The latter dictate that medical practice should become more standardized and evidence-based in order to optimize outcomes at the lowest cost. Current EHRs, mostly implemented over the past decade, are a first step in the digitization process, but do not support decision-making or streamline the workflow to the extent to which they are capable. In response, we propose a series of information system enhancements that we hope can be seized, improved upon, and incorporated into the next generation of EHRs.

There is already government support for these advances: The Office of the National Coordinator for Health IT recently outlined their 6-year and 10-year plans to improve EHR and health IT interoperability, so that large-scale realizations of this idea can and will exist. Within 10 years, they envision that we “should have an array of interoperable health IT products and services that allow the health care system to continuously learn and advance the goal of improved health care.” In that, they envision an integrated system across EHRs that will improve not just individual health and population health, but also act as a nationwide repository for searchable and researchable outcomes data [32]. The first step to achieving that vision is by successfully implementing the ideas and the system outlined above into a more fully functional EHR that better supports both workflow and clinical decision-making. Further, these suggested changes would also contribute to making the note writing process an educational one, thereby justifying the very significant time and effort expended, and would begin to establish a true learning system of health care based on actual workflow practices. Finally, the goal is to keep clinicians firmly in charge of the decision loop in a “human-centered” system in which technology plays an *essential but secondary role.* As expressed in a recent article on the issue of automating systems [33]:

In this model (human centered automation)…technology takes over routine functions that a human operator has already mastered, issues alerts when unexpected situations arise, provides fresh information that expands the operator’s perspective and counters the biases that often distort human thinking. The technology becomes the expert’s partner, not the expert’s replacement.

## Key Concepts and Terminology

A number of concepts and terms were introduced throughout this paper, and some clarification and elaboration of these follows:

- *Affordable Care Act (ACA)*: Legislation passed in 2010 that constitutes two separate laws including the *Patient Protection and Affordable Care Act* and the *Health Care and Education Reconciliation Act*. These two pieces of legislation act together for the expressed goal of expanding health care coverage to low-income Americans through expansion of Medicaid and other federal assistance programs [34].
- *Clinical Decision Support (CDS)* is defined by CMS as “a key functionality of health information technology” that encompasses a variety of tools including computerized alerts and reminders, clinical guidelines, condition-specific order sets, documentations templates, diagnostic support, and other tools that “when used effectively, increases quality of care, enhances health outcomes, helps to avoid errors and adverse events, improves efficiency, reduces costs, and boosts provider and patient satisfaction” [35].
- *Cognitive Computing* is defined as “the simulation of human thought processes in a computerize model…involving self learning systems that use data mining, pattern recognition and natural language processing to mimic the way the human brain works” [36]. Defined by IBM as computer systems that “are trained using artificial intelligence and machine learning algorithms to sense, predict, infer and, in some ways, think” [37].
- *Deep learning* is a form of machine learning (a more specific subgroup of *cognitive computing*) that utilizes multiple levels of data to make hierarchical connections and recognize more complex patterns to be able to infer higher level concepts from lower levels of input and previously inferred concepts [38]. Figure 3 demonstrates how this concept relates to patients illustrating the system recognizing patterns of signs and symptoms experienced by a patient, and then inferring a diagnosis (higher level concept) from those lower level inputs. The next level concept would be recognizing response to treatment for proposed diagnosis, and offering either alternative diagnoses, or change in therapy, with the system adapting as the patient’s course progresses.
- *Dynamic clinical data mining (DCDM)*: First, data mining is defined as the “process of discovering patterns, automatically or semi-automatically, in large quantities of data” [39]. DCDM describes the process of mining and interpreting the data from large patient databases that contain prior and concurrent patient information including diagnoses, treatments, and outcomes so as to make real-time treatment decisions [19].
- *Natural Language Processing (NLP)* is a process based on machine learning, or deep learning, that enables computers to analyze and interpret unstructured human language input to recognize and even act upon meaningful patterns [39,40].

## Abbreviations

ACA: Affordable Care Act
CDS: clinical decision support
CPG: clinical practice guidelines
DCDM: dynamic clinical data mining
EBM: evidence-based medicine
EHR: electronic health record
RCT: randomized control trial
SOAP: subjective, objective, assessment, and plan
